# Cross-sectional validation of a preoperative multidimensional assessment tool for third molar extraction using machine learning

**DOI:** 10.4317/medoral.28065

**Published:** 2026-04-19

**Authors:** Alba Sánchez Torres, Xavier Arias Huerta, Brais Pérez Iglesias, Elvira Gómez Reig, Rui Figueiredo, Eduard Valmaseda Castellón, J Alberto Conejero, Cosme Gay Escoda

**Affiliations:** 1 Department of Dentistry, Faculty of Medicine and Health Sciences, University of Barcelona, Spain. Oral and Maxillofacial Pathology and Therapeutics, IDIBELL Institute, Barcelona, Spain; 2 Oral Surgery and Implantology, Faculty of Medicine and Health Sciences, University of Barcelona, Spain; 3 Applied Mathematics Department, University Institut of Pure and Applied Mathematics (IUMPA), Universitat Politècnica de València, Spain; 4 Surgical, Oral and Maxillofacial Pathology, Faculty of Medicine and Health Sciences, University of Barcelona, Spain. Oral Surgery and Implantology (EFHRE International University/FUCSO). Oral and Maxillofacial Pathology and Therapeutics (IDIBELL Institute), Barcelona, Spain. Oral Surgery, Oral Implantology and Maxillofacial Surgery, Teknon Medical Center, Barcelona, Spain

**Keywords:** Learning curve, operative time, education, dental, machine learning, intraoperative complications, third molar.

## Abstract

**Background:**

This study aimed to assess the correlation between a preoperative difficulty assessment form and surgery time in third molar extractions. Secondary aims included analyzing the relationship between the form’s global score and surgical technique, surgeon-perceived difficulty, and postoperative complications. Machine learning models were also explored.

**Material and Methods:**

A cross-sectional study was conducted on patients requiring a third molar extraction between April 2022 and May 2023, operated by students of the master's degree in Oral Surgery and Implantology (University of Barcelona, Spain). Three previously calibrated investigators completed the preoperative difficulty evaluation form, which included clinical, radiological and surgical variables. Descriptive and bivariate analyses were performed using the Stata/IC 15.1. Machine learning models were applied to predict surgery time and surgeon-perceived difficulty.

**Results:**

A total of 205 patients, 75 males (36.6%) and 130 females (63.4%), with a mean age of 28.5±14.1 years were included; 49 (23.9%) were upper and 156 (76.1%) lower third molars. The global score of the form was significantly correlated to surgery time (Spearman's rho=0.640; P<0.001), perceived difficulty (Spearman's rho=0.395; P<0.001) and the surgical technique according to Parant’s classification (P<0.001). Postoperative complications were associated to higher scores on the difficulty evaluation form (P=0.012).

**Conclusions:**

The preoperative assessment form is a valid tool for estimating third molar extraction difficulty. Its global score is positively associated with surgery time, perceived difficulty, surgical technique, and postoperative complications. Machine learning models showed better performance at modeling surgery time than surgical difficulty.

## Introduction

Third molar extraction is one of the most common procedures in oral surgery [1,2]. Identifying predictive parameters for planning third molar surgery is essential to reduce postoperative morbidity and avoid intra- and postoperative complications [3-5].

According to the systematic review published by Sánchez-Torres *et al*. [1], there are several clinical, radiological, and surgical factors that can increase the difficulty of extraction. For example, the proximity of the roots to the maxillary sinus and to the inferior alveolar nerve (IAN) are factors associated with greater surgical difficulty, and are also related to a longer surgery time, since they require more precise surgical techniques [1].

Most prediction scales include radiological variables [4], which may reduce reliability in predicting surgical difficulty [6,7]. Gay-Escoda *et al*. [4] recently proposed a preoperative difficulty assessment form consisting of clinical, radiological and surgical variables to assess the ability of the dentist to estimate the difficulty, plan the surgical intervention or refer the patient to another more experienced professional, while considering possible risks and complications. To date, surgery time, the difficulty perceived by the surgeon at the end of the operation, and the type of surgical technique used have been the most commonly used variables to assess the difficulty of a surgical procedure [1,4].

The surgeon's experience is also included in the evaluation process. Currently, no standardized criteria exist for evaluating surgical experience, and while some studies measure it in terms of the years of professional practice, others measure it based on the number of operations performed [4]. Thus, the mentioned form takes into account the number of years of practice and also the degree of specialization of the professional. 

The principal hypothesis of the present study was that there is a positive correlation between surgical difficulty as assessed by the form and surgery time, and secondarily with the surgical technique, perceived difficulty, and the presence of complications.

The main objective of this study was to determine the correlation between the preoperative evaluation of surgical difficulty based on the form and the surgery time in patients requiring the extraction of upper or lower third molars. Likewise, this study aimed to determine the relationship between preoperative difficulty using the form and different clinical factors, such as the surgical technique, surgeon-perceived difficulty, and the occurrence of complications. In addition, the use of machine learning models was explored to evaluate their predictive capacity in relation to these variables.

## Materials and Methods

A cross-sectional study was conducted in candidates for upper or lower third molar extraction between April 2022 and May 2023. The operations were performed at the Dental Hospital of the University of Barcelona by students of the master degree in Oral Surgery and Implantology (University of Barcelona, Spain). This study adhered to the ethical principles outlined in the Declaration of Helsinki and was approved by the local Research Ethics Committee (CEIm Hospital Odontològic Universitat de Barcelona; protocol number 17/2022). All patients were informed and signed an informed consent to participate in the study. The study was conducted according to the STROBE (STrengthening the Reporting of OBservational studies in Epidemiology) statement [8].

*Participants*

The inclusion criteria were patients over 18 years of age requiring upper or lower third molar extraction and without relevant systemic disorders (American Society of Anesthesiologists [ASA] score I and II). The exclusion criteria were patients with acute pericoronitis or severe periodontal disease, patients under antibiotic or under any pharmacological treatment interfering with wound healing, and patients with contraindications to the local anesthesia. In the event of patients requiring the extraction of two third molars, only one was randomly selected.

*Surgical technique*

The extraction of third molars was performed under local anesthesia using 4% articaine and 1:100,000 epinephrine (Artinibsa; Inibsa, Lliçà de Vall, Spain). The removal of upper third molars started with the buccal and palatal administration of the anesthetic solution, while in the case of lower third molars, inferior alveolar nerve blocks and buccal infiltrative anesthesia were employed. The surgical technique for the extraction of erupted third molars involved syndesmotomy, luxation, avulsion, alveolar curettage and irrigation. In the case of impacted third molars, the surgical technique involved a triangular flap with a mesial vertical release incision, bone removal with a low-speed handpiece, sectioning of the crown and/or roots (in the case of lower third molars), luxation and avulsion with dental elevators and/or forceps, and suturing with 3/0 polyglactin 910 (Vicryl®, Ethicon, Inc., USA).

The postoperative medication consisted of an antiinflammatory drug (ibuprofen, Normon® 600 mg, one tablet every 8 hours for 3-5 days), an analgesic (paracetamol, Normon®, 1 g, one tablet every 8 hours, if required) and rinses with 0.12% chlorhexidine (PerioAid, Dentaid®, Cerdanyola del Vallès, Spain, 15 mL every 12 hours for 7 days, starting the day after the operation). Antibiotics were only prescribed when the surgeon considered that the patient had a high risk of postoperative infection (usually amoxicillin, Normon® 750mg, one tablet every 8 hours). 

The patients were informed of the expected signs and symptoms in a normal postoperative period so that they could detect the development of any complication. Patients were visited on the seventh postoperative day for suture removal and to record possible complications.

*Study variables*

The surgical experience of the master degree student and the score of the preoperative difficulty assessment form were recorded. This form has been already published [4] and is composed of clinical, radiological, and surgical variables. It allows each clinical case to be classified into three categories of difficulty. Each item is scored as 1 point for low difficulty, 2 points for moderate difficulty, and 5 points for great difficulty. A global score of under 20 indicates low difficulty, a score of 20-40 indicates moderate difficulty, and a score of over 40 indicates great difficulty. 

Variables indicating surgical difficulty were also recorded, such as surgery time (measured in minutes from the beginning of the incision or syndesmotomy to the last suture), the surgical technique according to Parant’s classification [9], postoperative difficulty as reported by the surgeon using a visual analogue scale (0-100 mm), and intraoperative (bleeding, oroantral communication, displacement of the tooth into anatomical cavities) and postoperative complications (maxillary sinusitis, nerve injury, postoperative infection and alveolar osteitis).

*Calibration*

Three investigators (BP, XA and EGR) were calibrated by evaluating the Winter, and Pell & Gregory classifications of 10 cases not included in the sample. Inter-examiner agreement was determined by the kappa index, obtaining a value of >0.7.

*Sample calculation*

Sample calculation was performed using the Stata/IC 15.1 statistical package (StataCorp LLC, Lakeway Drive, TX, USA). An estimate of a proportion was made for a finite population of 2000 patients, based on a known proportion of complex cases of 23.8%, defined by a surgery time of over 40 minutes [5]. For an absolute precision of ±4% and a 95% confidence level, 358 subjects were needed.

*Statistical analysis*

The study data were processed using the Stata/IC 15.1 statistical package (StataCorp LLC, Lakeway Drive, TX, USA). The normal distribution of the main variable (form score) was tested using the Shapiro-Wilk test, and descriptive and bivariate analyses of the data were performed using parametric tests (Student t-test for scale variables or one-way analysis of variance [ANOVA]) or non-parametric tests in the case of a non-normal distribution (Mann-Whitney U-test or Kruskal-Wallis test) to compare means in order to relate the total score of the form to surgical difficulty as measured according to surgery time, the surgical technique, and difficulty reported by the surgeon. In addition, the association of surgical difficulty with the occurrence of intraoperative and postoperative complications was determined. Finally, the association between the result of the preoperative assessment form and surgery time, as well as difficulty reported by the surgeon, was also assessed. Statistical significance was set at *p<*0.05.

On the other hand, a complementary analysis was performed using machine learning models based on decision trees, with the aim of exploring the predictive potential of these techniques in relation to the variables "surgery time" and "difficulty reported by the surgeon". In particular, the Random Forest (RF) and XGBoost [10] algorithms were used, selected for their ability to generate more accurate predictions compared to traditional methods such as Lasso and Ridge regression. These models also allow identification of the variables with the greatest impact on the result, thus providing a more complete view of the factors determining the difficulty of extraction. This approach aims to illustrate the usefulness of recent artificial intelligence techniques in clinical contexts, even when a limited dataset is available, as is in the present study [10].

## Results

A total of 205 patients, 75 men (36.6%) and 130 women (63.4%), with a mean age of 28.5±14.1 years were included in the study. A total of 26 extractions involved a surgery time of over 40 minutes, indicating surgical procedures that can be considered to involve great difficulty. The estimated statistical power was calculated and, considering the recorded 12.7% of difficult cases with respect to the known proportion of complex cases of 23.8% reported in the literature [5] used for the sample size calculation, a power of 98% was obtained. Patient recruitment was therefore considered to have been completed.

Of the 205 extracted third molars, 49 (23.9%) were upper molars and 156 (76.1%) were lower molars. Eighty-one extractions (39.5%) were performed by first-year students, 105 (51.2%) by second-year residents, and 19 (9.3%) by third-year students.

The distribution of the global score of the preoperative difficulty form was found to be non-normal (Shapiro-Wilk test =0.002) and presented negative skewness. Likewise, surgery time and surgeon-perceived difficulty also failed to meet normality (*P<*0.001).

The distribution of items checked on the form is shown in Table 1. 

Table 2 shows the distribution and bivariate analysis between the outcome variables (global form score, surgery time, perceived difficulty and surgical technique) and the students’ experience. An increased surgical complexity was observed when more experienced students were involved. The global score presented a significant correlation with surgery time (Spearman's rho=0.640; *P<*0.001) and perceived difficulty (Spearman's rho=0.395; *P<*0.001), indicating that the form adequately measured surgical difficulty.

The bivariate analysis presented in Table 2 shows the relationship among the different categories according to Parant’s classification [9] (*P<*0.001).

Regarding the intraoperative complications, 7 patients (3.4%) who underwent lower third molar extraction experienced bleeding that was resolved with local measures. On the other hand, 8 patients (3.9%) experienced postoperative complications: four patients (2%) had an inferior alveolar nerve injury, and 5 patients (2.4%) suffered a postoperative infection (4 lower third molar extractions and 1 upper third molar removal). One patient presented both complications.

Table 3 shows the bivariate analysis results between the presence of complications and the global form score, in addition to surgeon experience and surgery time. Specifically, postoperative complications were associated with greater difficulty according to the evaluation form score (*P=*0.012). Longer surgery time was significantly associated with the occurrence of intraoperative (*P=*0.024) and postoperative (*P=*0.045) complications; however, there was no difference regarding surgeon experience.

The dataset with the difficulty assessment form and outcome variables (surgery time and surgeon-reported difficulty) were considered for establishing the machine learning model. To contrast the relevance of the variables, several datasets were used: Reduced dataset, reduced dataset adding the variable time and reduced dataset adding the variable VAS score. To train the models, each dataset was divided into 80% for training and evaluation and 20% for prediction, using a maximum of 200 estimators and 5 cross-validations. Table 4 shows the mean squared error (MSE) and the coefficient of determination R^2^ for indicating how the model adequately accounts for the variability of the data, as well as the variables with more weight in the explanation of the model.

The models demonstrated that objective variables were more effective in predicting surgery time than subjective surgeon-reported difficulty. Furthermore, models predicting surgery time required fewer trees, making them more generalizable for limited datasets. Figure 1 illustrates the importance of the variables for each prediction.

## Discussion

The global score of the form was found to be significantly correlated with surgery time as well as with perceived difficulty, indicating that it adequately measures surgical difficulty. Thus, the form proposed by Gay-Escoda *et al*. [4] can be regarded as a reliable tool for the preoperative assessment of the surgical difficulty of third molar extraction. Likewise, a statistically significant relationship was observed between the student’s experience and higher scores on the form, surgery time, perceived difficulty, and the type of surgical technique. This suggests that the challenging procedures were performed by more experienced surgeons. The statistical analysis was intentionally based on bivariate associations, as the main objective of the study was to evaluate the convergent validity of the form. Operating time, surgical technique, and perceived difficulty by the surgeon were considered indicators related to surgical difficulty.

On the other hand, the presence of complications was not related to surgeon experience. However, longer operations had a higher likelihood of developing intraoperative and postoperative complications. Extractions with higher scores in the global form were also more prone to postoperative complications. 

The use of machine learning techniques such as Random Forest and XGBoost allowed us to identify variables with a great impact on the preoperative difficulty of third molar extraction. Although this approach does not replace conventional methods, it provides an additional perspective, especially valuable when seeking to establish clinically relevant predictive models. Despite the small sample size involved, the tree-based models proved capable of providing strong and consistent predictions, suggesting that these tools may be useful even in settings characterized by limited data. These results open the door to future research involving larger datasets, where the use of artificial intelligence could optimize clinical decision making and further personalize surgical planning.

In the educational context, the studied form can be useful to assess the students' learning curves and to encourage surgeons to perform adequate preoperative planning. The Dunning-Kruger concept explains that people who lack skills in a particular area tend to overestimate their own abilities and, conversely, those who achieve better results tend to underestimate themselves [11-13]. This miscalibration is associated with a deficit in metacognitive ability or the ability to distinguish accuracy from error. Surprisingly, improving participants' skills, and therefore increasing their metacognitive competence, helps to recognize the limitations of their abilities, which in turn, is one of the prerequisites for self-improvement, and the acquisition of new knowledge and skills [13].

Thus, the learning curve is the time required and/or the number of procedures necessary for a novice surgeon to be able to perform the operation independently and with reasonable results [14]. The curve depends on the surgeon's skills as well as on acquired knowledge and patient factors such as complex anatomy [14]. The studied form allows determination of the starting point and to observe the progress of the practitioner's skills. It can also promote lifelong learning for practicing clinicians, enabling continuous improvement and skill refinement [15].

The surgeon’s level of experience is a determinant of surgical difficulty [1] and is essential in both the planning process and the decision to refer the patient to a specialist [4]. One of the main limitations of this study is that the form was tested in surgeons with a similar profile (postgraduate students of an Oral Surgery and Implantology master's degree program). Although the students had slightly different degrees of experience (first, second- and third-year residents), the sample was quite homogenous. This aspect limits the external validity of the study, as the results cannot be directly extrapolated to a population of surgeons with different levels of experience. Therefore, it is necessary to perform additional research involving surgeons with different profiles, training, and degrees of experience to confirm these findings. Patient anxiety, another relevant factor when assessing surgical difficulty, was not objectively measured using validated questionnaires. Instead, a subjective evaluation was performed, as the form under study is designed for use in routine clinical settings and should be completed quickly and intuitively.

Increased surgical experience is generally associated with reduced stress levels. Transitioning from theoretical knowledge to clinical practice can be demanding and may induce significant stress in novice surgeons. A high degree of stress can impair the learning process, and hinder decision making and clinical performance, which can affect treatment and ultimately patient safety. On the other hand, a moderate degree of stress enhances concentration, increases alertness, and improves the consolidation of skills [16]. Therefore, assessment of the surgeon's degree of stress and its influence on surgical difficulty could be a future line of research. 

This study has some limitations that should be mentioned. First, neither the assessment form nor the machine learning models were externally validated, so the results should be interpreted with caution, and subsequent studies in other samples are required. The study did not directly compare the form with common difficulty scales, although the authors considered that some of the variables included in pre-existing radiological scales were already included in the form. Furthermore, the limited number of upper third molars limits the generalisation of the results for this subgroup. It should be noted that machine learning models have only been used as an exploratory tool given the limited dataset. Future longitudinal studies using multivariate models are required to confirm the predictive capacity of the form.

## Conclusions

The preoperative assessment form evaluated in this study demonstrated good reliability and showed significant associations with surgery time, surgeon-perceived difficulty, and the surgical technique employed in third molar extractions. Additionally, higher scores were associated with an increased incidence of postoperative complications.

Within the limitations of a cross-sectional design, the machine learning models demonstrated increased accuracy and robustness in modeling surgery time compared to surgeon-reported difficulty, supporting their usefulness for exploratory analyses in small datasets. Furthermore, the form may serve as a useful educational resource for monitoring students’ learning curves and promoting structured preoperative planning. 

## Figures and Tables

**Table 1:** Table 1: Distribution of responses on the preoperative evaluation form regarding the surgical difficulty of upper or lower third molar extraction. Results presented as frequencies and proportions.

		**Low difficulty**		**Moderate difficulty**		**High difficulty**	
**Clinical features**							
*Age*		<25 years	106 (51.7)	25-50 years	83 (40.5)	>50 years	16 (7.8)
*Body mass index (BMI) (kg/m* ^2^ *)*		<25	141 (69.1)	25-30	47 (23.1)	>30	16 (7.8)
*Ethnicity*		Caucasian	182 (88.8)	Asiatic	20 (9.8)	African	3 (1.5)
*Systemic disease*		ASA I	187 (91.2)	ASA II	15 (7.3)	ASA III and IV	3 (1.5)
*Anxiety level*		Not anxious	139 (67.8)	Anxious but cooperative	56 (27.3)	Phobic- uncooperative	10 (4.9)
*Facial pattern*		Dolichocephalic	49 (23.9)	Mesocephalic	125 (61)	Brachycephalic	31 (15.1)
*Mouth opening*		>45mm	81 (39.5)	35-45mm	120 (58.5)	<35mm	4 (2)
**Radiological variables**							
*Root morphology*		Conical fused roots	91 (44.4)	Multi-radicular (≥2 roots)	96 (46.8)	Germ Bulbous roots Dilacerated roots	18 (8.8)
*Distal space available*		Pell and Gregory I	113 (55.1)	Pell and Gregory II	80 (39)	Pell and Gregory III	12 (5.9)
*Depth*		Pell and Gregory A	100 (48.8)	Pell and Gregory B	79 (38.5)	Pell and Gregory C	26 (12.7)
*Angulation*		Mesio-angulated	67 (32.7)	Vertical	84 (41)	Horizontal	34 (16.6)
						Disto-angulated	20 (9.7)
*Proximity to anatomical structures*	*Maxillary sinus*	Apex not in contact with cortex	19 (43.2)	Apex in contact with cortex	14 (31.8)	Apex overlapping or exceeding cortex	11 (25)
	*Inferior alveolar nerve*	Apex distal to upper cortex of the lower dental canal	48 (30.8)	Apex overlapping with upper cortex	81 (51.9)	Overlapping or loss of both cortices	17 (10.9)
						Narrowing of the duct	10 (6.4)
				Darkening of roots	42 (26.9)	Duct deviation	9 (5.8)
						Bent apex	0
*Relationship to the second molar*		Absent	53 (25.9)	The tooth contacts the crown of the second molar.	83 (40.5)	The tooth contacts the root and/or crown of the second molar	69 (33.7)
*Periodontal ligament*		Radiolucent	158 (77.1)	Mixed	37 (18.1)	Radiopaque	10 (4.8)
**Surgical variables**							
*Anesthesia*		No pathological history	201 (98)	Intolerance to vasoconstrictor	3 (1.5)	Previous anesthetic failure	1 (0.5)
*Degree of impaction*		Erupted	70 (34.1)	Semi-erupted Partial bone impaction	132 (64.4)	Intraosseous	3 (1.5)
*Surgical technique*		Conventional extraction with forceps and dental elevators	89 (43.4)	Need for ostectomy	43 (21)	Ostectomy and tooth sectioning	73 (35.6)
*Presence of associated lesions*		Absence of lesions Thickened follicle	188 (91.7)	Associated lesion <10mm	16 (7.8)	Associated lesion >10mm	1 (0.5)
*Surgeon experience*		Senior dentist with experience in oral surgery > 10 years	-	Dentist with Master Degree or formal training in oral surgery >3 years	-	General dentist	-
				General dentist with specific continuing training in oral surgery >5 years	-	Supervised student	205 (100)

**Table 2:** Table 2: Descriptive and bivariate analysis between outcome variables and surgical experience. Data reported as the mean (standard deviation). † Kruskal-Wallis test. ‡ Chi-square test. * Surgical technique (Parant scale); 1: Extraction with forceps and elevators only. 2: Extraction by ostectomy. 3: Extraction by ostectomy and coronal section. 4: Complex procedures [9].

**Course**		**Descriptive**	**1** ^st^ ** year resident**	**2** ^nd^ ** year resident**	**3** ^rd^ ** year resident**	*P-* **value**
Global form score		37.1 (7.8)	31.4 (4.5)	40.3 (7.4)	43.8 (5.9)	<0.001†
Surgery time		23.3 (15.9)	16.7 (14)	26.2 (15)	36 (16.5)	0.001†
Perceived difficulty		3.6 (2)	3.2 (2)	3.7 (1.8)	4.4 (2.3)	0.026†
Surgical technique*	1	81 (39.5)	60 (74.1)	20 (19.1)	1 (5.3)	<0.001‡
	2	46 (22.4)	13 (16.1)	31 (29.5)	2 (10.5)	
	3	43 (21)	6 (7.4)	29 (27.6)	8 (42.1)	
	4	35 (17.1)	2 (2.5)	25 (23.8)	8 (42.1)	

**Table 3:** Table 3: Bivariate analysis between the presence of intraoperative and postoperative complications, and the global score of the form, surgery time and surgeon experience. † Kruskal-Wallis test. ‡ Chi-square test. SD: Standard deviation.

	**Global form score** †		**Surgery time (min)** †		**Surgeon experience** ‡		
	**Mean** **(SD)**	*P-* **value**	**Mean (SD)**	*P-* **value**	**Year of** **residency**	**Operations (%)**	*P-* **value**
**Intraoperative complications**							
No	37 (7.8)	0.410	22.7 (15.4)	0.024*	1^st^	80 (39)	0.279
					2^nd^	100 (48.8)	
					3^rd^	18 (8.8)	
Yes	39.7 (8)		40.9 (21.3)		1^st^	1 (0.5)	
					2^nd^	5 (2.4)	
					3^rd^	1 (0.5)	
**Postoperative complications**							
No	36.8 (7.7)	0.012*	22.9 (15.6)	0.045*	1^st^	80 (39)	0.379
					2^nd^	99 (48.3)	
					3^rd^	18 (8.8)	
Yes	45 (8.6)		34.9 (19.1)		1^st^	1 (0.5)	
					2^nd^	6 (2.9)	
					3^rd^	1 (0.5)	

**Table 4:** Table 4: Analysis of the predictions of the variables time and VAS score from Random Forests and XGBoost.

**Predicted variable ** **and model**	**Number of best** **model estimators**	**R** ^2^	**Variables with greater impact in the model**	
**Surgery time** **(Random Forests)**	60	0.62625	Surgical technique	0.650245
			Age	0.060982
			Root morphology	0.049780
			Ethnicity	0.032542
			Impaction of the third molar	0.023904
			Facial pattern	0.017587
**Surgery time** **(XGBoost)**	200	0.58182	Surgical technique	0.257121
			Periodontal ligament	0.056443
			Ethnicity	0.056252
			Overlapping or loss of the two cortical layers of the inferior alveolar nerve	0.043464
			Body mass index	0.043170
			Age	0.043066
**Difficulty reported by the surgeon (Random Forests)**	60	0.456658	Technique	0.380642
			Distal space	0.063595
			Age	0.059152
			Proximity to the maxillary sinus	0.058856
			Relation to the second molar	0.055807
			Anxiety	0.052926
**Difficulty reported by the surgeon (XGBoost)**	200	0.431059	Surgical technique	0.138652
			Depth	0.051938
			Age	0.051382
			Distal space available	0.050176
			Overlapping or loss of the two cortical layers of the inferior alveolar nerve	0.049407
			Facial pattern	0.046817

**Figure 1:**
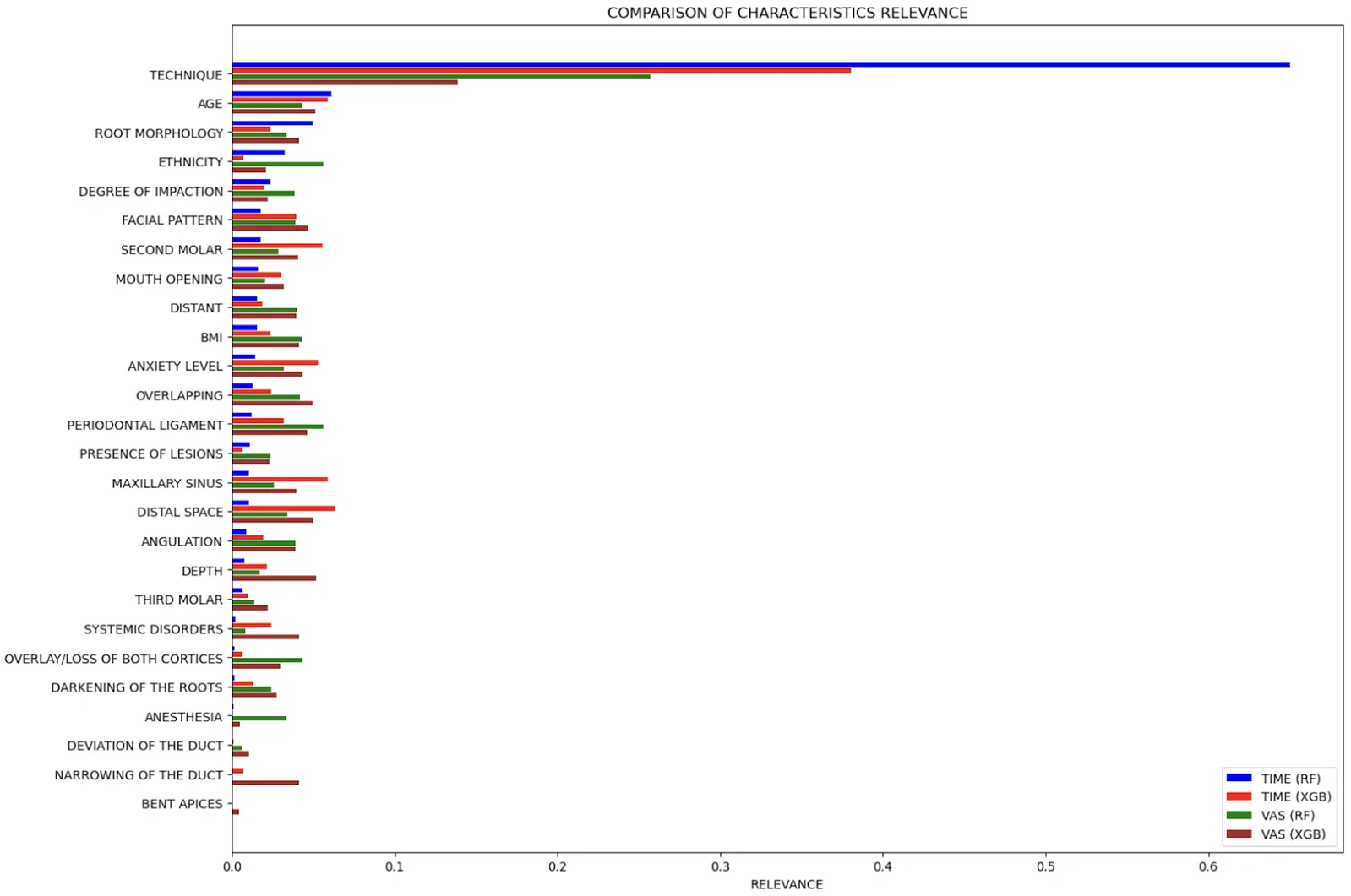
Fig. 1: Analysis of the most important variables for predicting surgery time and surgeon-reported difficulty from Random Forests and XGBoost.

## Data Availability

None.
